# Self-compassion is associated with the superior longitudinal fasciculus in the mirroring network in healthy individuals

**DOI:** 10.1038/s41598-023-39384-z

**Published:** 2023-07-28

**Authors:** Yeong-Geon Hwang, Chongwon Pae, Chae Rim Song, Hyun-Ju Kim, Minji Bang, Chun Il Park, Tai Kiu Choi, Min-Kyoung Kim, Sang-Hyuk Lee

**Affiliations:** 1 Department of Psychiatry, CHA Bundang Medical Center, CHA University, 59 Yatap-ro, Bundang-gu, Seongnam-si, Gyeonggi-do 13496 Republic of Korea; 2 Department of Psychiatry, CHA Ilsan Medical Center, CHA University, 1205, Jungang-ro, Ilsandong-gu, Goyang-si, Gyeonggi-do 10414 Republic of Korea; 3 Graduate School of Clinical Counseling Psychology, CHA University, Seongnam-si, Republic of Korea

**Keywords:** Neuroscience, Psychology

## Abstract

Self-compassion (SC) involves taking an emotionally positive attitude towards oneself when suffering. Although SC has positive effects on mental well-being as well as a protective role in preventing symptoms in healthy individuals, few studies on white matter (WM) microstructures in neuroimaging studies of SC has been studied. Brain imaging data were acquired from 71 healthy participants. WM regions of mirroring network were analyzed using tract-based spatial statistics. After the WM regions associated with SC were extracted, exploratory correlation analysis with the self-forgiveness scale, the coping scale, and the world health organization quality of life scale abbreviated version was performed. We found that self-compassion scale total scores were negatively correlated with the fractional anisotropy (FA) values of the superior longitudinal fasciculus (SLF) in healthy individuals. The self-kindness and mindfulness subscale scores were also negatively correlated with FA values of the same regions. These FA values were negatively correlated with the total scores of self-forgiveness scale, and self-control coping strategy and confrontation coping strategy. Our findings suggest levels of SC may be associated with WM microstructural changes of SLF in healthy individuals. These lower WM microstructures may be associated with positive personal attitudes, such as self-forgiveness, self-control and active confrontational strategies.

## Introduction

Self-compassion (SC) describes an emotionally positive attitude extended toward ourselves when we suffer, consisting of three main components; self-kindness, common humanity, and mindfulness^1^. SC entails being warm and understanding towards ourselves when encountering pain or personal shortcomings, rather than ignoring them or flagellating ourselves with self-criticism. SC also involves recognizing that suffering and failure are part of the shared human experience rather than isolating. In addition, SC requires taking a mindful approach to one’s feelings and thoughts, without judgment of them.

Recent studies showed that psychological interventions utilizing SC may be effective in relieving students’ depressive symptoms^2,3^, and preventing postpartum depression in mothers for up to one year following childbirth^4^. SC may contribute not only to the prevention of depressive symptoms, but also to improving the overall quality of life such as health, social relationships, and the environment^5,6^. SC showed a significantly positive association with adaptive coping strategies, such as positive reinterpretation or acceptance, and a negative association with maladaptive strategies such as rumination or avoidance^7,8^. Self-forgiveness can be categorized as an adaptive emotional coping strategy for stress related to guilt or shame. Self-forgiveness entails accepting one’s wrong and responsibility while replacing negative thoughts and feelings with compassion^9^. A structural equation model has shown that SC is associated with low levels of self-punitiveness and high levels of self-forgiveness^10^. In addition, the relationship between SC and general health appears to be mediated by self-forgiveness^11^. Despite the important protective roles of SC, its neurobiological mechanisms have not yet been clearly identified, and neuroimaging research has been yet insufficient. Furthermore, it has never been investigated whether SC-related brain regions are associated with other psychological characteristics, such as self-forgiveness, coping strategies, and quality of life.

SC is inversely correlated with gray matter volume (GMV) in the left dorsolateral prefrontal cortex (DLPFC), and its mindfulness component is associated with greater GMV in the dorsomedial prefrontal cortex, anterior cingulate cortex (ACC) and left supplementary motor area^12^. The results suggested that SC may be associated with brain regions involved in self-referential and emotion processing^12^. These regions overlap with the default mode network (DMN), which are the major neurobiological basis of depression and anxiety disorders related to self-processing and mind-wandering^13,14^. Recent neuroimaging studies have demonstrated brain connectivity between the frontal region or DMN and other brain regions, conceptualizing neural networks of self-related and social cognitive processing based on their specific connectivity^15^. Among them, mirroring network cannot be left out, which is a system associated with neurocognitive functions such as social cognition, empathy, and theory of mind^16,17^.

In humans, putative mirroring network is formed from collection of regions including the inferior frontal gyrus (IFG), inferior parietal lobule (IPL), superior temporal sulcus, anterior insula, ACC, and amygdala^16–19^. It is known that SC can be strongly associated with compassion for others in a general pattern^1,20,21^. Previous functional neuroimaging studies reported that self-critical thought was associated with activity in DLPFC and ACC, and self-reassuring thought with activity in temporal pole and insula^22^. In addition, SC was positively associated with activity in IPL and insular during sad face recognition among healthy individuals^23^. These results may suggested that generating SC involves the similar processes as generating compassion or empathy for others^22^, and that SC can be associated with empathy-related regions such as mirroring network^23^.

As we mentioned above, a study showed the GMVs of the prefrontal cortex, ACC, and supplementary motor area related to social cognition such as empathy were correlated with SC^12^; however, there has been no study about brain white matter (WM) connectivity. Wang, et al.^24^ extensively reviewed the literature describing WM tracks in social brain networks and defined that the superior longitudinal fasciculus (SLF), inferior longitudinal fasciculus (ILF), inferior fronto-occipital fasciculus (IFOF), anterior thalamic radiation (ATR), and uncinate fasciculus (UF) are all included in the mirroring network. Fractional anisotropy (FA) values in these regions were commonly associated with empathy in healthy individuals^25^. Based on these backgrounds, we hypothesized that mirroring network can be related to levels of SC in healthy individuals. Therefore, the present study investigates brain WM connectivity in the mirroring network in relation to SC in healthy individuals, additionally exploring whether WM alterations in these regions are related to SC related factors such as self-forgiveness, coping strategies, and quality of life.

## Results

### Socio-demographics and clinical characteristics

The socio-demographics and clinical characteristics of the participants are summarized in Table 1. Among the 71 participants, 41 were female and 30 were male, and almost all participants had received a bachelor's degree or higher; more detailed descriptions are presented in Table 1.

**Table 1:** Socio-demographics and clinical characteristics of 71 study participants were presented.

	Mean ± SD or N (%)
Age at scan (year)	37.61 ± 8.28
Sex	
Female	41 (57.75%)
Male	30 (42.25%)
Years of education (year)	17.34 ± 1.98
Intracranial volume (ml)	1526.98 ± 130.95
Self-Compassion Scale (SCS)	3.48 ± 0.39
Self-kindness	2.64 ± 0.65
Self-judgment	1.81 ± 0.65
Common humanity	2.74 ± 0.88
Isolation	1.64 ± 0.60
Mindfulness	3.07 ± 0.73
Over-identification	2.10 ± 0.71
Self-forgiveness scale (SFS)	72.08 ± 8.09
Coping scale	
Confrontation	9.40 ± 3.24
Distancing	3.62 ± 2.73
Self-control	6.64 ± 3.35
Seeking social support	7.74 ± 3.49
Accepting responsibility	4.43 ± 2.01
Escape–avoidance	8.40 ± 2.69
Planned problem solving	5.78 ± 2.42
Positive reappraisal	10.16 ± 3.75
WHOQOL-BREF	
Overall quality of life	3.57 ± 0.70
General health	3.39 ± 0.83
Physical health domain	15.28 ± 1.86
Psychological domain	14.15 ± 2.18
Social relationships domain	14.19 ± 2.27
Environmental domain	14.31 ± 2.29

Abbreviations: *SD* Standard Deviation, *N (%)* subject number (percent), *WHOQOL-BREF* WHO Quality of Life Scale Abbreviated version.

### Relationship between the scores of self-compassion and the mirroring network

**Figure 1:**
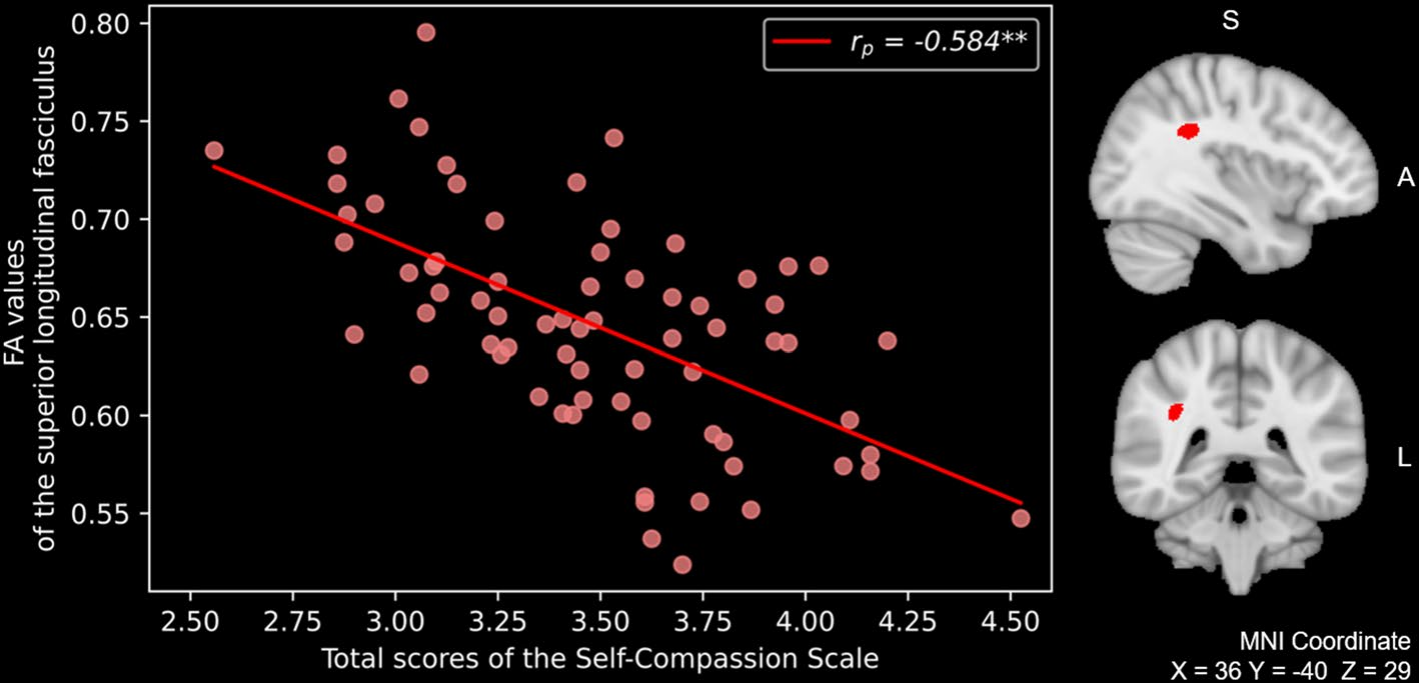
The total scores of SCS were negatively correlated with FA values of the right superior longitudinal fasciculus in healthy individuals [*p* < 0.05 (FWE-corrected)]. Images of the sagittal and coronal view were superimposed on the MNI 1 mm template. For better visibility, the result was thickened using the “tbss-fill” command. *SCS* self-compassion scale, *FA* fractional anisotropy, *FEW* Family-Wise Error, *S* superior, *A* anterior, *L* left, *MNI* Montreal Neurologic Institute.

A voxel-wise correlation analysis was performed between the self-compassion scale (SCS) total scores and diffusion tensor imaging (DTI) measures of the mirroring network. The SCS total scores showed a significant negative correlation with FA values from the right SLF [*p* < 0.001 (Family-Wise Error, FWE –corrected); Fig. 1]. Additionally, radial diffusivity (RD) values were positively correlated in the same region [*p* = 0.023 (FWE-corrected)], whereas mean diffusivity (MD) and axial diffusivity (AD) were not significantly correlated. Sex, age at the time of the magnetic resonance imaging (MRI) scan, and intracranial volume (ICV) were all included as covariates, and did not change the significance level of the correlations.

**Figure 2:**
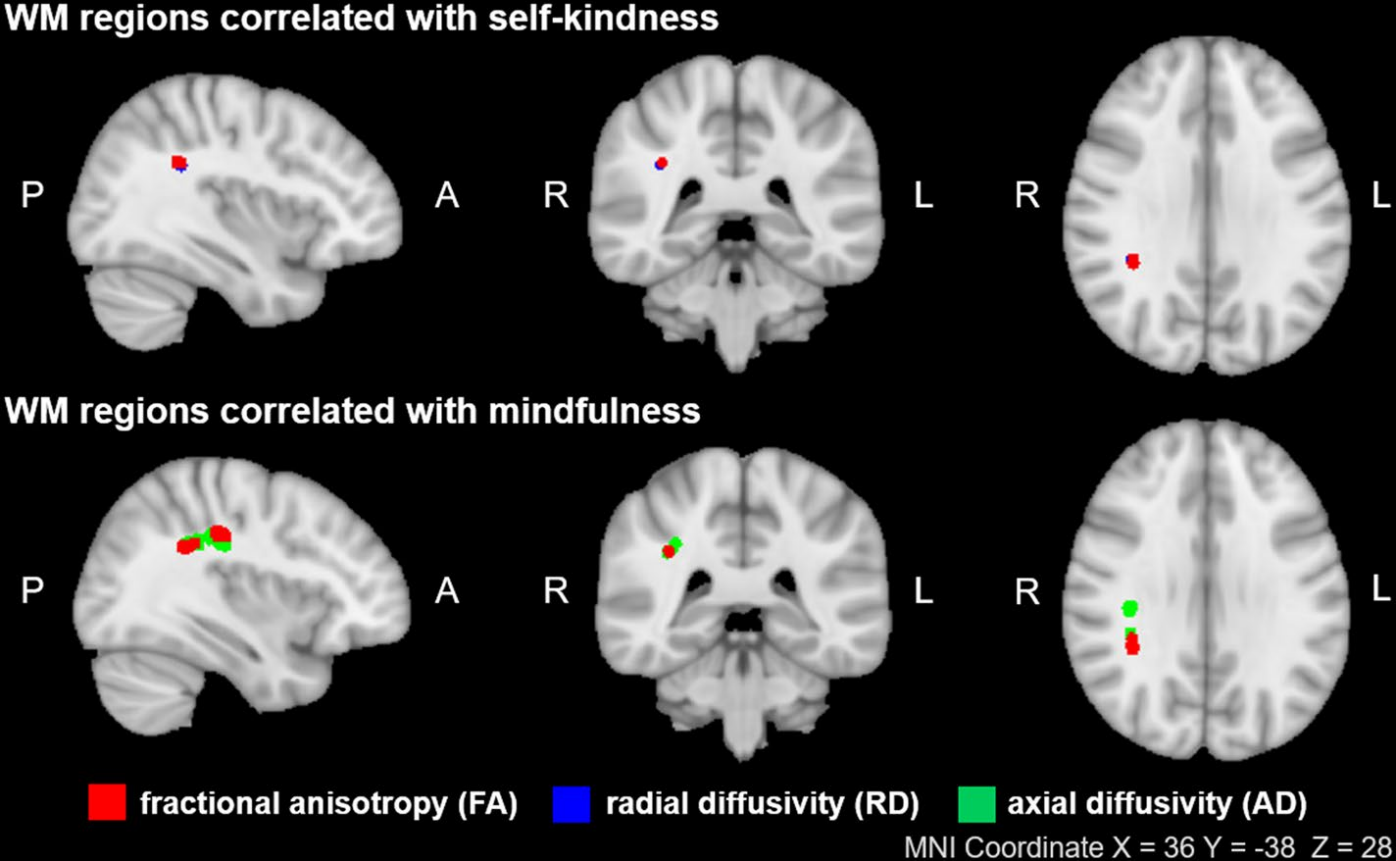
Voxel-wise correlations between the self-kindness and mindfulness subscale scores of the self-compassion scale and the FA (red), AD (green) and RD (blue) values of the superior longitudinal fasciculus are presented [*p* < 0.05 (FWE-corrected)]. The results were thickened using the “tbss-fill” command and superimposed on the MNI1mm template. *WM* white matter, *FA* fractional anisotropy, *RD* radial diffusivity, *AD* axial diffusivity, *P* posterior, *A* anterior, *R* right, *L* left, *MNI* Montreal Neurologic Institute.

The self-kindness and mindfulness subscale scores of the SCS were negatively correlated with FA values of the right SLF [*p* = 0.035, *p* = 0.006 (FWE-corrected), respectively; Fig. 2], but the other subscales did not show a significant correlation. Significant associations were maintained after controlling for sex, age and ICV as covariates.

The self-kindness subscale scores showed a positive correlation with the RD values of the right SLF [*p* = 0.044 (FWE-corrected); Fig. 2], and the mindfulness subscale scores showed a negative correlation with the AD values of the right SLF [*p* < 0.001 (FWE-corrected); Fig. 2]. Significance remained after controlling for covariates. No significant correlations were found with other DTI measures.

### Exploratory correlation analysis between FA values of the SLF and scores of self-forgiveness, coping strategies, and quality of life

**Figure 3:**
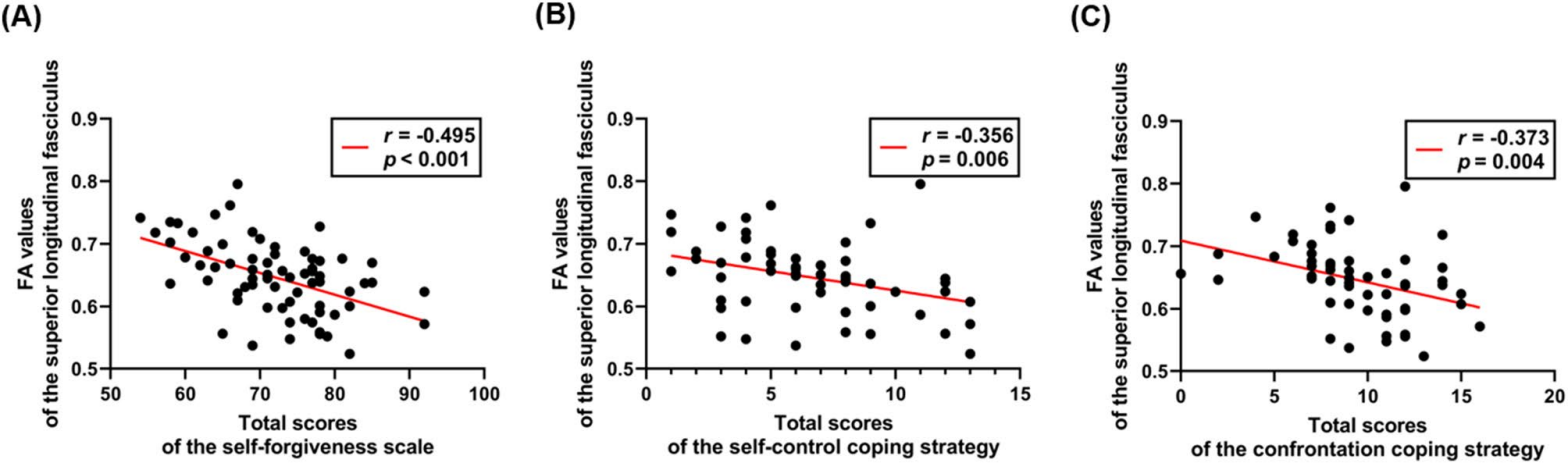
Scatter plots show the Pearson exploratory correlations between the FA values of the superior longitudinal fasciculus and total scores of the other assessments (FDR < 0.05): (**A**) self-forgiveness scale (**B**) self-control coping strategy (**C**) confrontation coping strategy. *FA* fractional anisotropy, *FDR* false discovery rate.

The FA values of the right SLF showed a significant negative correlation with total self-forgiveness scale (SFS) total scores (*r* =  − 0.495, *p* < 0.001; Fig. 3a), self-control (*r* =  − 0.356, *p* = 0.006; Fig. 3b), and confrontation (*r* =  − 0.373,* p* = 0.004; Fig. 3c) coping strategy scores. The *p*-values in the correlations survived false discovery rate (FDR) corrections (FDR < 0.05). FA values were not significantly correlated with the four-domain scores of the World Health Organization quality of life scale (WHOQOL-BREF), and only the general health item scores showed a trend toward significance (*p* = 0.074). They are reported here as exploratory results for use as predictions in future studies.

## Discussion

We found that the SCS total scores were correlated with decreased FA values in the SLF in healthy individuals. Moreover, the self-kindness and mindfulness subscale scores, which are positive components of SCS, were correlated with decreased FA values in the same regions. The SLF regions were correlated with the total scores of SFS and coping strategies which use self-control and confrontation.

In our study, the SLF regions were found to be associated with levels of SC in healthy individuals. These SLF regions were the second and third branches of the SLF (SLF II and SLF III) in the right parietal region. SLF II and III connect the caudal and rostral part of the IPL, respectively, to the prefrontal cortex^26,27^. The IPL may represent a brain region that is one node of both the mirroring network and DMN, and both networks can process self-relevant information^15^. Previous studies have shown that activation of DMN regions, including IPL, is related to the mind-wandering^28^ and rumination^29^ as well as self-referential processing of episodic memory^30^.

Correspondingly, recent neuroimaging studies have suggested that the high FA values in the right SLF and the hyper-connectivity of the right caudal part of the IPL are associated with insomnia severity and poor sleep quality in insomnia patients^31,32^. In addition, the FA values of the right SLF have been shown to be positively associated with false memory recall^33^, which can be usually associated with mind-wandering or self-referential processing. Studies on loving kindness meditations found that meditators showed a relatively deactivated caudal and rostral part of the IPL compared with non-meditators, and deactivations were interpreted as reducing mind wandering^34,35^. Furthermore, stronger functional connectivity between the IPL and IFG showed highly divergent thinking, which may be more involved in mind-wandering^36^. Based on these results, we suggested that high FA values in SLF may be negatively associated with mindful approach ability.

Our data suggested that the association between SC and WM microstructures in the SLF can also be associated with the self-kindness and mindfulness subscales. We found that higher SCS total scores or its self-kindness or mindfulness subscale scores were associated with lower FA/lower AD/higher RD values in the SLF. FA, which is a measure of microstructural integrity, could be a sensitive detecting microstructural changes, but does not provide specificity regarding the type of changes^37^. Changes in myelination can cause an increase in RD with a small decrease in AD^37^. Taken together, changes in microstructure or myelination in SLF may be associated with SC in healthy individuals.

Among healthy participants, we found that the FA values of the SLF regions were negatively correlated with positive personal attitudes such as self-forgiveness, self-control and confrontational coping strategies. SC may be positively associated with self-forgiveness, and low shame partially mediates this relationship^11^. Moreover, Bzdok, et al.^38^ suggested that brain activity during moral cognition and mind wandering might overlap in the DMN. Previous brain studies have found that self-compassion were negatively associated with the ACC and DLPFC, which might indicate that SC was related to self-regulation^12,22^. Brewer, et al.^34^ suggested that reduced mind wandering may be involved in the increased connectivity between the DMN and self-control regions of the brain. We emphasize that correlation analyses between FA values of SLF regions and scale scores were exploratory in nature, so confirmation will be needed in future planned studies.

Some limitations should be considered. First, this was a cross-sectional study that measures the brain WM connectivity in relation to trait of SC in healthy individuals. Although it was measured based on the stable trait of SC^39,40^, our measurement may be limited in that it does not accurately reflect the current experience of SC in the subjects. In order to clarify the results, future studies will be needed to measuring the state of SC currently being experienced^41^, or considering whether or not the SC practice has been experienced recently should be performed. Second, the sample size was relatively small^42^, and exact pre-power calculation was not performed. In the latest studies of the highly cited structural MRI study at the time, the median value of the sample size was 50^43^, and the analysis was conducted by collecting data from more than 70 participants. Future studies with larger sample sizes and accurate pre-calculations should be performed to confirm our results. Third, since exploratory correlation analyses between the FA values of WM and other psychological characteristics (self-forgiveness, coping strategies, and quality of life) were performed without sufficient hypotheses in nature, future planned studies will require confirmation of these results. Furthermore, this was a correlation analysis that could not clarify whether psychological characteristics could cause a relationship between SC and FA values in the SLF regions.

## Method

### Participants

Seventy-one neurologically and psychiatrically healthy participants were enrolled from the local community. Participants were recruited from the Department of Psychiatry at CHA Bundang Medical Center of CHA University (Gyeonggi-do, Republic of Korea) through advertisements from January 2014–September 2021. All participants were aged between 23 and 61 years, were Korean, and right-handed. Exclusion criteria for all participants included any current or past history of neurological disorders, intellectual disabilities, traumatic brain injury or major psychiatric disorders including anxiety, mood, or psychotic disorders. All study procedures complied with the regulations of the Institutional Review Board (No. 2019-05-030, 2021-03-001) of CHA Bundang Medical Center. After sufficient information about the study was provided to the participants, written informed consent was acquired in accordance with the 1964 Helsinki Declaration and its corresponding updates, as well as Good Clinical Practice guidelines.

### Procedures

A total of 71 participants underwent MRI on a GE Signa HDxt 3.0 T MRI scanner, supplied by GE Healthcare (Milwaukee, Wisconsin, USA) with an eight-channel phased-array head coil at CHA Bundang Medical Center. Using an echo planar imaging (EPI) sequence, diffusion-weighted imaging was performed as follows: repetition time = 17,000 ms, echo time = 108 ms, field of view = 240 mm, matrix = 144 × 144, slice thickness = 1.7 mm, and voxel size = 1.67 × 1.67 × 1.7 mm^3^. To reduce current related distortions and the effects of EPI spatial distortions, a double echo option, an eight-channel head coil, and an array of spatial sensitivity encoding with a speed-up factor of two were used. Seventy axial slices parallel to the anterior commissure-posterior commissure line encompassing the entire brain were obtained in 51 directions with *b* = 900 s/mm^2^. Eight baseline scans were performed with b0 = 0 s/mm^2^.

### Measures

The SCS was developed by Neff^44^ to measure overall levels of SC and is composed of six components: self-kindness, self-judgment, sense of common humanity, isolation, mindfulness, and over-identification. The questionnaire consists of 26 items each scored on a 5-point scale, ranging from *almost never* (1) to *almost always*—(5). To assess levels of SC, we used the Korean version of the SCS, whose Cronbach’s alpha was 0.90 for the total score, but ranged from 0.74 to 0.81 for the six subscale scores^45^. Scores for the negative subscales (i.e., self-judgment, isolation, and over-identification) were reverse-coded, and the SCS total score calculated by averaging the six subscale means. The SCS total score indicated the degree of overall self-compassion.

The SFS was also used^46^. This questionnaire comprises 19 items, each rated on a 1–5 point scale, with three factors: acceptance and improvement, responsibility, and negative affect, thought, and behavior. A higher SFS total score indicates a higher degree of self-forgiveness. The SFS had high reliability, with an alpha of 0.87^46^.

The coping scale developed by Folkman, et al.^47^ was used to measure coping strategies employed during stressful situations. The scale comprises 50 items scored on a 5-point scale. This scale consisted of eight subscales: confrontation, distancing, self-control, seeking social support, accepting responsibility, escape avoidance, planned problem solving, and positive reappraisal. A previous study showed a reliability range from 0.50 to 0.89^48^.

The WHOQOL-BREF is a 26-item measure of quality of life. We used the Korean version of the WHOQOL-BREF, which consists of four domains: physical health, psychological health, social health, and environmental health, and two facets: overall quality of life and general health^49^. The Cronbach’s alpha of the Korean version was high, ranging from 0.58 to 0.78 for each domain and 0.90 for the total score^49^.

### Diffusion tensor imaging analysis

Voxel-wise statistical analysis of diffusion data was performed using Tract-Based Spatial Statistics (TBSS, v1.2), implemented in the FMRIB Software Library (FSL v6.0.5, Oxford, UK, https://fsl.fmrib.ox.ac.uk/fsl), according to standard procedure^50^. FA was used to investigate the brain WM connectivity based on diffusion MRI. FA values can be obtained through diffusion imaging and are a widely used indicator of WM connectivity. FA has a characteristic that increases as the diffusion direction of the water molecule is biased (i.e. anisotropic). DTI preprocessing which included skull stripping using the Brain Extraction Tool (BET) and eddy current correction was performed using FSL^51^. All participants’ FA data were aligned into standard space (Montreal Neurologic Institute, MNI 152 standard) for statistical analysis.

All transformed FA images were integrated and applied to the original FA map to create a standard space version of it. All transformed FA images were averaged to generate a mean FA image, which was then skeletonized to create a mean FA skeleton, considering the centers of the WM tracks. The threshold of the skeleton was set to FA > 0.2 to contain only major fiber bundles. Other DTI scalar measures (MD, AD and RD) were prepared in a similar manner according to the non-FA processing pipeline in the FSL.

**Figure 4:**
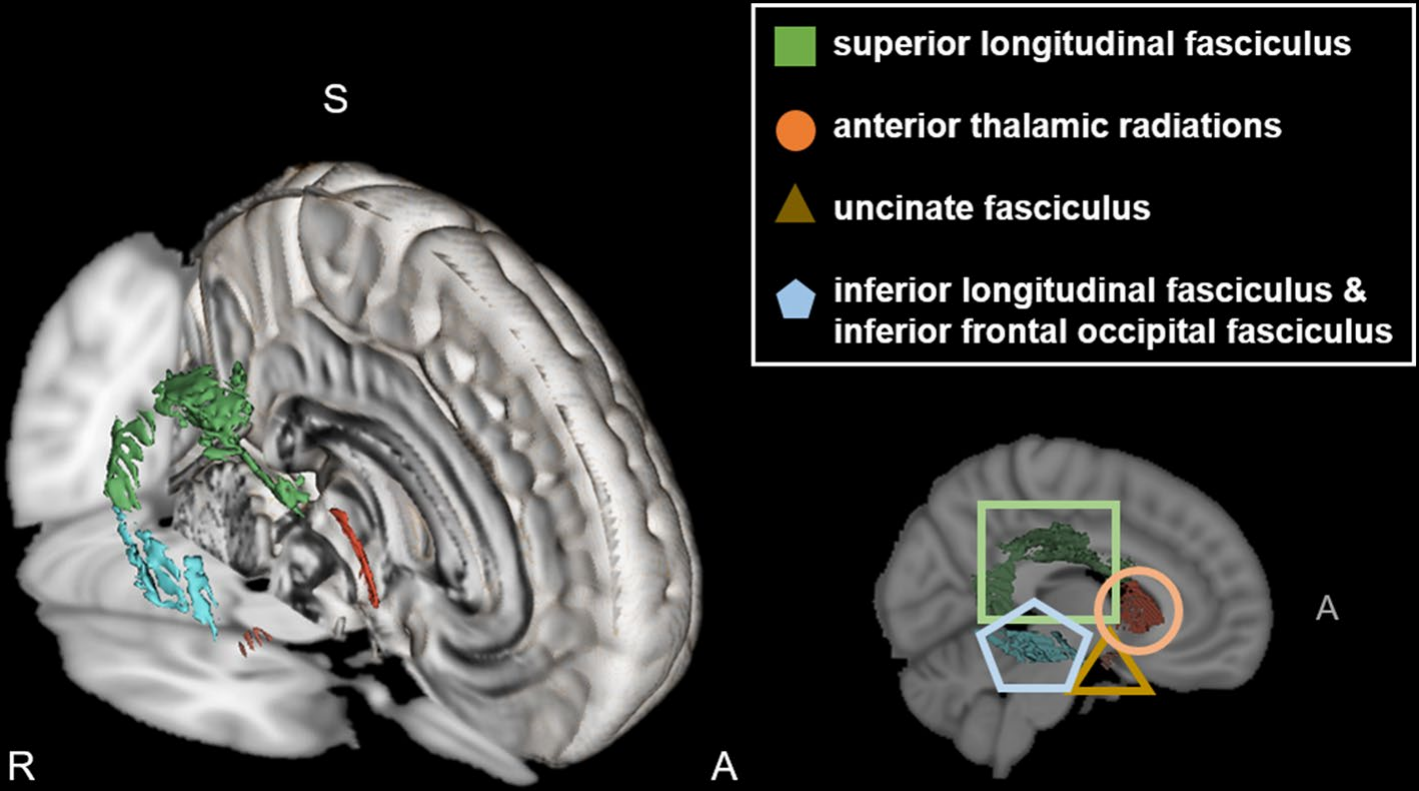
Using 3D Slicer, bilateral white matter regions in the mirroring network were extracted. The superior longitudinal fasciculus (green, square), anterior thalamic radiations (orange, circle), uncinate fasciculus (brown, triangle), and inferior longitudinal fasciculus and inferior fronto-occipital fasciculus (blue, pentagon) were selected as mirroring network. *A* anterior, *S* superior, *R* right.

The mirroring network regions of interest (ROIs) proposed by Wang, et al.^24^ were selected from the Johns Hopkins University (JHU) DTI-based probabilistic tractography atlas^52^. We extracted the following WM ROIs using 3D Slicer version 4.11^53^: the SLF, ILF, IFOF, ATR edited from the anterior limb of the internal capsule^54^, and UF (Fig. 4). The ROI mask was created by multiplying the mean FA skeleton with the regional mask of the WM underlying the mirroring network. We performed voxel-wise correlation analysis within the ROI mask by computing 10,000 permutations using Randomise in FSL. Multiple comparisons were corrected using the threshold-free cluster enhancement (TFCE) method. The threshold level was set at* p* < 0.05, corrected for FWE rate.

### Statistical analysis

Statistical analyses were performed using the Statistical Package for the Social Sciences (SPSS) 27.0.1 (IBM Corp., Armonk, NY, USA). To analyze the demographic information of healthy individuals, descriptive statistics were used for mean values and standard deviations.

To determine association between the level of SC and neural WM correlates, we performed voxel-wise correlation analysis between the SCS total scores and the FA, MD, AD, and RD values. We further analyzed the correlation between each SCS subscale score and DTI measures in the same manner. Sex, age, and ICV were set as the covariates. Sex and age were set as covariates as variables can be affect levels of self-compassion^55,56^ and WM changes^57^, and ICV was generally selected in the WM study. In addition to the main effects, each covariate was added to all TBSS analyses to control for other effects on brain structure and levels of self-compassion.

Following the results of the voxel-wise analysis, mean FA values were extracted from regions with a significant correlation with SCS total scores among the WM of the mirroring network. Exploratory correlation analysis was performed to determine the relationship between FA values of the extracted regions and the SFS, coping scale, and WHOQOL-BREF. Since correlation analysis was considered to be exploratory in nature, analysis was applied without sufficient hypotheses. Significance level was set at α = 0.05 for statistical significance. Furthermore, a FDR correction was performed (FDR < 0.05) to control for multiple correlation comparisons.

### Ethical approval

All procedures were performed after review and approval by the Institutional Review Board of the CHA Bundang Medical Center. After the participants had a detailed explanation of the study, written informed consent was obtained from the latest version of the Declaration of Helsinki. Further, the principles of Good Clinical Practice were acquired.

### Consent to participate

Informed consent was obtained from all individual participants included in the study.

## Data Availability

The datasets generated and analyzed during the current study are not publicly available due to legal or ethical restrictions that protect patients’ privacy and consent but available from the corresponding author on reasonable request.
